# Early Diagnosis of the Backward Curvature of the Spine

**Published:** 1866-11

**Authors:** Julien S. Sherman

**Affiliations:** Chicago


ARTICLE XLI.

### EARLY DIAGNOSIS OF THE BACKWARD CURVATURE OF THE SPINE.

BY JULIEN S. SHERMAN, M.D., Chicago.

The importance of diagnosing this disease in its insipiency must be apparent to all. Yet, in a large majority of cases, the difficulty is not suspected until the projection of one or more of the spinous processes reveal the fact, that the disease has already advanced to a change of structure and that a portion of the bodies of the vertebræ have undergone inflammatory softening, followed by absorption, and in many the cavity of an abscess formed.

The early recognition becomes much more important, when the fact is considered, that, the deformity once produced, we can do but little toward restoring the normal form of the spine. It remains a permanent change in its flexibility and symmetry. The arrest of its increase is the object to be accomplished by treatment.

The disease may, properly, be divided into two stages:—

1st. Simple inflammation of the intervertebral cartilages.

2d. Caries, suppuration, and deformity.

Very seldom is the inflammation of tubercular origin, but traumatic, following some injury to the spine, occasioned by a fall or blow upon the back, or violent flexion.

The age of the patient will also assist us in diagnosis; the disease being almost entirely confined to an early age, varying from one to eight years, during which period the spine is yet in the process of development and more liable to injury from slight causes.

Boys are more often the subjects of its attacks than girls, the reason of which is not obvious.

At the commencement of the first stage, the child will be noticed to play less vigorously than has been its custom, its motions are not as free, and there is an appearance of stiffness to the back, and an effort to remove the pressure from the inflamed tissues by resting the elbows on a table or chair, and supporting the shoulders and chest. When reaching to grasp articles from the floor, it will endeavor to accomplish the same object by placing one hand on the knee.

The inflammation, once started and aggravated by motion and pressure, slowly increases. The child now complains of pain, the position of which will aid us in determining what portion of the spine is affected. Seldom, in this stage, is there pain in the back—if present, it is deep seated, and tenderness cannot be detected by manipulation—but it is confined to the stomach and bowels; also radiating on the sides. It is not of a severe or spasmodic kind, but dull and persistent. These symptoms are often attributed to disorders of the digestive apparatus. They will be distinguished by the condition of the tongue and secretions, which, in this stage of spinal disease are not affected. Very rarely, the cervical region is involved, and the pain then is situated in the shoulders and sides of the neck. Difficulty of deglutition and disturbed respiration are sometimes experienced, although no special disease of these organs can be detected. The sleep of the patient is also disturbed, and the pulse increased in frequency.

If, in a period ranging from one to eight months from the commencement of the disease, the back be carefully examined, it will show, in some portion of its extent, a *bulging;* not a sharp *angular* projection, but a rounded prominence. The inflammation is yet limited to the cartilages and has not involved the bones. The projection is, consequently, produced by the approximation of the bodies of several vertebræ, which continues the curve over a larger portion of the spine and gives it the oval appearance.

Soon, the sharp projection of one of the spinous processes of the vertebræ makes its appearance, and the characteristic sharp angle is formed. It has now advanced to the second stage and the deformity has begun. The inflammation has proceeded to the body of the vertebra corresponding to the projecting process; softening and absorption of its structure has taken place, allowing the bodies of the vertebræ above and below to approximate more closely, and give the appearance of a backward dislocation of the diseased one. The destruction is now more rapid, and caries and suppuration soon follow. The membranes of the spinal cord, and sometimes the cord itself, becoming involved, partial paralysis of the spinal muscles often ensues, causing one shoulder to drop, and giving the patient a sidewise motion in walking. If confined to the lumbar region, the same pathological condition causes reflex contractions of the flexor muscles of the thigh, which may be mistaken for hip-disease. The differential diagnosis is, however, easy. There is no pain or tenderness in the hip-joint, and none referred to the knee, so characteristic in that disease.

The angle is sometimes projected laterally, simulating lateral curvature. It may be distinguished from this by its abruptness and sharpness. The lateral form being an even and rounded curve, as it advances it assumes more the backward direction.

The symptoms in the first stage are sufficiently characteristic, that a close observer will not fail to detect the disease. We can seldom err in our diagnosis, when we find them following an injury; and only on their early recognition and prompt treatment can deformity be avoided.

